# Safety of bronchoscopy in patients with malignant hematologic disorders

**DOI:** 10.1186/s12890-020-01283-8

**Published:** 2020-09-11

**Authors:** Hironori Uruga, Toshitaka Sato, Aya Nishida, Naoyuki Uchida, Masanori Tsuji, Shuhei Moriguchi, Yui Takahashi, Kazumasa Ogawa, Kyoko Murase, Shigeo Hanada, Hisashi Takaya, Atsushi Miyamoto, Nasa Morokawa, Muneyoshi Kimura, Hideki Araoka, Rumiko Tsuchihashi, Yuki Asano-Mori, Atsushi Wake, Shuichi Taniguchi, Kazuma Kishi

**Affiliations:** 1 Department of Respiratory Medicine, Respiratory Center, Toranomon Hospital, Tokyo, Japan; 2 Department of Respiratory Medicine, Toranomon Hospital Kajigaya, Kawasaki, Kanagawa Japan; 3 Okinaka Memorial Institute for Medical Research, Tokyo, Japan; 4 Respiratory Center, KKR Sapporo Medical Center, Sapporo, Japan; 5 Department of Hematology, Toranomon Hospital, Tokyo, Japan; 6 Department of Hematology, Toranomon Hospital Kajigaya, Kawasaki, Kanagawa Japan; 7 Department of Infectious Diseases, Toranomon Hospital, Tokyo, Japan; 8 Department of Respiratory Medicine, Toho University Omori Medical Center, Tokyo, Japan

**Keywords:** Bronchoscopy, Acute myeloid leukemia, Myelodysplastic syndrome, Malignant lymphoma, Hematopoietic stem cell transplantation, Midazolam

## Abstract

**Background:**

Factors affecting the safety of bronchoscopy in patients with malignant hematologic disorders have not been well described**.** We evaluated the safety of bronchoscopy and describe factors affecting its complication rate in such patients.

**Methods:**

Between January 2009 and December 2018, 316 bronchoscopies in 282 patients with malignant hematologic disorders and pulmonary infiltrates were performed at our institution. The bronchoscopic procedure used and its complications were evaluated.

**Results:**

The most common underlying disease was acute myeloid leukemia (134/282 patients, 47.5%). Platelet transfusion was performed the day before or the day of bronchoscopy in 42.4%, supplemental oxygen was administered before the procedure in 23.1%, and midazolam was used in 74.4%. Thirty-five bronchoscopies (11.1%) were complicated by hemoptysis and 7 patients developed pneumothorax, 4 of whom required thoracic drainage. Two patients (0.6%) were intubated within 48 h of the procedure and prolonged oxygen desaturation (> 48 h) occurred in 3.8%. Multivariate analysis showed that only use of midazolam significantly reduced the risk of prolonged oxygen desaturation (hazard ratio 0.28, 95% confidence interval 0.09–0.85, *p* = 0.03). Transbronchial lung biopsy significantly increased the risk of hemoptysis (hazard ratio 10.40, 95% confidence interval 4.18–25.90, *p* = 0.00), while use of midazolam significantly reduced the risk (hazard ratio 0.31, 95% confidence interval 0.14–0.73, *p* = 0.01).

**Conclusions:**

Bronchoscopy is relatively safe in patients with malignant hematologic disorders. Caution and judicious use of sedatives may improve the patient’s procedural tolerance and lower complications.

## Background

Pulmonary complications occur in up to 40–60% of patients with hematologic diseases and have a considerable influence on morbidity and mortality [1]. These complications include infiltration of underlying disease, opportunistic infections, and various pulmonary infiltrates after hematopoietic stem cell transplantation (HSCT), such as idiopathic pneumonia syndrome. Several studies have demonstrated the utility of bronchoscopy in patients with hematologic disorders [2–6], but the factors affecting the safety of bronchoscopy in such patients have not been well described. Here, we evaluated the safety of bronchoscopy at our institution and its complication rate with the aim of elucidating these factors.

## Methods

### Subjects

The institutional review board of Toranomon hospital approved this study (No. 1845). This retrospective study involved patients with malignant hematologic disorders and pulmonary infiltrates who underwent bronchoscopy between January 2009 and December 2018 in a fully equipped endoscopy room in the Department of Respiratory Medicine, Toranomon Hospital, Tokyo, Japan. Bronchoscopies performed in another ward or in the intensive care unit were excluded from the analysis. The need for bronchoscopy was discussed at weekly multidisciplinary team meetings that included hematologists, infectious diseases specialists, and pulmonologists. Bronchoscopy was contraindicated in patients with severe disseminated intravascular coagulation or bleeding tendency. The bronchoscopic procedure used and its complications were evaluated.

### Bronchoscopy procedure

Chest computed tomography was performed using an Aquilion 16 or 64 system (Toshiba Medical Systems, Otawara, Japan), and platelet transfusion was performed for patients with platelet count ≤20 × 10^3^/μl before bronchoscopy whether we conducted transbronchial lung biopsy (TBLB) or not. The bronchoscopic procedures were performed using a 4.9-mm bronchoscope (LTF-260; Olympus, Tokyo, Japan). All patients inhaled 5 mL nebulized 1% lidocaine solution before the procedure, and lidocaine was squirted in the airways during the procedure. Use of midazolam for sedation during bronchoscopy at our institution was started primarily from the beginning of 2011, which was in the middle of the study period. We administered midazolam 0.03 mg/kg intravenously before bronchoscopy and usually added 0.01 mg/kg once or twice during the procedure as needed. Before and after starting to use midazolam, there was no change in bronchoscopist skill or in the approach to patients and overall patient care in the periprocedural period. All bronchoscopies were oral and all procedures were performed by pulmonologists. Oxygen was trans nasally supplied during procedure, if necessary. Bronchoalveolar lavage was performed using 100–150 mL of normal saline at room temperature and bronchial washing was performed with 10–50 mL of saline. All patients were placed in the supine position for at least 1 h after biopsy before undergoing chest radiography. The attending physician then evaluated the severity of pneumothorax and the need for placement of a chest tube.

### Statistical analysis

Oxygen desaturation was defined as worsening of oxygen level compared with that at the time of entering the endoscopy room. Prolonged oxygen desaturation was defined as an oxygen level continuously below baseline for more than 48 h. Univariate analysis was performed using Fisher’s exact test and the Mann-Whitney *U* test and multivariate analysis using multiple logistic regression. Variables with a *p*-value < 0.2 in the univariate analysis were entered into the multivariate analysis by the variable increase method. All statistical analyses were performed used SPSS statistical software (version 18.0, IBM Corp., Armonk, NY).

## Results

A total of 207 men and 75 women of median age 61 years were included in this study. Bronchoscopy was performed twice in 30 patients and 3 times in 2 patients, giving a total of 316 bronchoscopies in 282 patients. The most common underlying disease was acute myeloid leukemia (AML) including myelodysplastic syndromes (MDS)-overt AML (*n* = 134, 47.5%). One hundred and thirty-two patients had undergone HSCT (cord blood transplantation, *n* = 69; unrelated transplantation [*n* = 35] or related [*n* = 1] bone marrow transplantation; autologous [*n* = 15] or related [*n* = 12] peripheral blood stem cell transplantation). Median platelet count was 91,000/μL (3000–568,000) in all patients, 126,000/μL (9000–561,000) in patients who underwent TBLB and 64,000/μL (3000–568,000) in those who did not undergo TBLB. Platelet transfusion was performed the day before or the day of the bronchoscopy in 134 procedures (42.4%). Median neutrophil count was 2840/μL. Supplemental oxygen was administered before bronchoscopy at a rate of 0.5–6 L/min in 73 bronchoscopies (23.1%). Bronchial washing was included in 201 bronchoscopies (63.6%), bronchoalveolar lavage in 104 (32.9%), TBLB in 125 (39.6%), bronchial curettage in 6 (1.9%), and endobronchial ultrasound-guided transbronchial needle aspiration in 2 (0.6%). Pethidine was used in 261 bronchoscopies (82.6%), midazolam in 235 (74.4%), and atropine sulfate in 291 (92.1%).

**Fig. 1:**
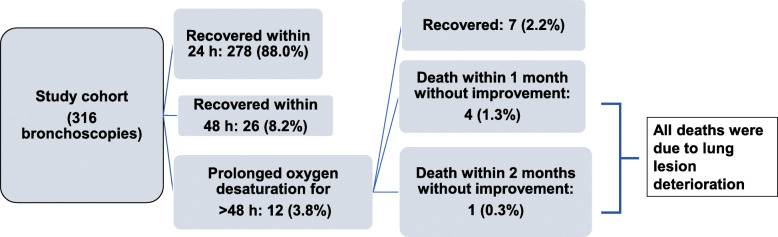
Flowchart of patients with the complication of oxygen desaturation after bronchoscopy

Thirty-five bronchoscopies (11.1%) were complicated by hemoptysis and 7 patients developed pneumothorax, 4 of whom required thoracic drainage. There were no severe cardiovascular complications such as arrhythmia and hypotension requiring vasopressor. Two patients (0.6%) were intubated within 48 h of the procedure. The first patient was intubated on the day after the procedure and died the following month; the second patient was intubated just after the procedure and underwent extubation the following day (Table 1). The oxygen desaturation data are summarized in Fig. 1. Prolonged oxygen desaturation (> 48 h) occurred after 12 of the 316 bronchoscopies (3.8%). Oxygen desaturation did not recover in 5 of these 12 patients and was ultimately fatal. The data for these 12 patients are summarized in Table 1. Hemoptysis was found in 3 of the 12 patients, but none developed pneumothorax. Oxygen desaturation recovered in 7 patients (including 2 with organizing pneumonia, 2 with invasion of underlying disease, and 1 with pneumocystis pneumonia). The 5 patients with no recovery of oxygen desaturation included 2 patients with aspergillosis and 2 with cytomegalovirus pneumonia.

**Table 1:** Summary of 12 patients with prolonged oxygen desaturation (> 48 h) after bronchoscopy

Case	Underlying disease	Sedation	Platelet (× 103/μl)	Oxygen before bronchoscopy	Oxygen after bronchoscopy	Method	Clinical diagnosis	Hemoptysis	Outcome
1	ALL	Fentanyl 3 mg/hour	37	RA	1 L/min	Bronchial wash	Aspergillosis	–	Died after 2 months
2	AML	Peth 27.2 mg	20	RA	1 L/min	Bronchial wash	Unknown	–	Died after 3 weeks
3	AML	Peth 20.9 mg	9	RA	4 L/min	Bronchial wash	CMV pneumonia	+	Died after 9 days
4	ALL	Peth 18.5 mg	110	6 L/min	12 L/min	Bronchial wash	CMV pneumonia	–	Intubated, died after 1 month
5	AML/MDS	HH 25 mg	48	1 L/min	1 L/min	Bronchial wash	Aspergillosis	+	Died after 13 days
6	AML	Peth 25 mg	161	2 L/min	8 L/min	Bronchial wash	OP	–	Recovered
7	AML/MDS	Mid 1.5 mg, Peth 28 mg	38	RA	2 L/min	Bronchial wash	Underlying disease	–	Recovered
8	AML	Peth 27.3 mg	25	RA	RA	Bronchial wash	OP	–	Recovered
9	ALL	Peth 21 mg	384	RA	10 L/min	BAL, TBLB	Pneumocystis pneumonia	+	Intubated, recovered
10	AML	Mid 1.2 mg, Peth 27.0 mg	14	1 L/min	3 L/min	Bronchial wash	Unknown	–	Recovered
11	AML	Mid 1.5 mg, Peth 26.3 mg	98	RA	3 L/min	Bronchial wash	Underlying disease	–	Recovered
12	AML	Mid 2 mg, Peth 31.5 mg	171	RA	5 L/min	Bronchial wash	Aspergillosis	–	Recovered

*ALL* Acute lymphocytic leukemia, *AML* Acute myeloid leukemia, *BAL* Bronchoalveolar lavage, *CBT* Cord blood transplantation, *CMV* Cytomegalovirus, *HH* Hydroxyzine hydrochloride, *MDS* Myelodysplastic syndromes, *Mid* Midazolam, *OP* Organizing pneumonia, *Peth* Pethidine, *RA* Room air, *rBMT* Related BMT, *rPBST* Related peripheral blood stem cell transplantation, *TBLB* Transbronchial lung biopsy, *uBMT* Unrelated bone marrow transplantation

Univariate and multivariate analyses were performed to identify risk factors for prolonged oxygen desaturation (> 48 h) after bronchoscopy. Factors identified as statistically significant in univariate analysis were platelet count ≤20 × 10^3^/μl, TBLB not performed, and midazolam not administered (Table 2). The multivariate analysis showed that only use of midazolam significantly reduced the risk of prolonged oxygen desaturation (hazard ratio 0.28, 95% confidence interval 0.09–0.85, *p* = 0.03; Table 2).

**Table 2:** Risk factors for prolonged oxygen desaturation

Characteristic	Prolonged oxygen desaturation group (*n* = 12)	Improvement group (*n* = 304)	*p* value of univariate analysis	*p* value in multivariate analysis	Hazard ratio (95% CI)
Age ≤ 70 years	0	39	0.37		
Sex (male/female)	10/2	225/79	0.74		
AML and MPAL	9	153	0.09	0.19	
Platelet count (≤20 × 103/μl)	**3**	**26**	**0.05**	0.12	
Platelet transfusion	7	127	0.26		
Neutrophil count (≤500)	2	61	1.00		
Within 180 days from BMT except PBSCT	3	58	0.71		
Inhalation of oxygen (≥5 L/min)	1	5	0.10	0.31	
Bronchial wash	9	192	0.55		
BAL	3	101	0.76		
TBLB	**2**	**119**	**0.14**	0.15	
**Use of midazolam**	**5**	**230**	**0.01**	**0.03**	**0.28 (0.09–0.85)**
Use of pethidine	10	251	1.00		
Use of atropine sulfate	10	281	0.24		

Bold indicates statistical significance

*AML* Acute myeloid leukemia, *BAL* Bronchoalveolar lavage, *BMT* Bone marrow transplantation;

*MPAL* Mixed phonotype acute leukemia, *PBST* Peripheral blood stem cell transplantation, *TBLB* Transbronchial lung biopsy

Univariate analysis for hemoptysis shows TBLB was the only significant risk factor. Multivariate analysis showed that TBLB significant increased the risk for hemoptysis (hazard ratio 10.40, 95% confidence interval 4.18–25.90, *p* = 0.00; Table 3), while use of midazolam significantly reduced the risk (hazard ratio 0.31, 95% confidence interval 0.14–0.73, *p* = 0.01; Table 3).

**Table 3:** Risk factors for hemoptysis

Characteristic	Patients with hemoptysis (*n* = 35)	Patients without hemoptysis (*n* = 281)	*p* value of univariate analysis	*p* value in multivariate analysis	Hazard ratio (95% CI)
Age ≤ 70 years	3	36	0.60		
Sex (male/female)	29/6	206/75	0.22		
AML and MPAL	17	145	0.74		
Platelet count (≤20 × 103/μl)	1	28	0.23	**0.69**	
Platelet transfusion	17	117	0.43		
Neutrophil count (≤500)	9	54	0.36		
Within 180 days from BMT except PBSCT	4	57	0.21		
Inhalation of oxygen (≥5 L/min)	0	6	1.00		
Bronchial wash	15	164	0.26		
BAL	10	79	0.59		
**TBLB**	28	93	**0.00**	**0.00**	**10.40 (4.18–25.90)**
**Use of midazolam**	22	213	0.10	**0.01**	**0.31 (0.14–0.73)**
Use of pethidine	32	259	0.75		
Use of atropine sulfate	30	231	0.61		

Bold indicates statistical significance

*AML* Acute myeloid leukemia, *BAL* Bronchoalveolar lavage, *BMT* Bone marrow transplantation;

*MPAL* Mixed phonotype acute leukemia, *PBST* Peripheral blood stem cell transplantation, *TBLB* Transbronchial lung biopsy

## Discussion

We performed 316 bronchoscopies in 282 patients with malignant hematologic disorders and pulmonary infiltrates over a period of 10 years. Acute myeloid leukemia accounted for about half of the underlying diseases. Two patients were intubated within 48 h of the procedure and 12 experienced prolonged oxygen desaturation. Multivariate analysis showed that use of midazolam significantly reduced the risk of prolonged oxygen desaturation by approximately a quarter, whereas TBLB increased the risk of hemoptysis approximately 10-fold.

A review by Harris et al. [6] identified the following complications of bronchoscopy after HSCT: bleeding (1.5–15%), pneumothorax (0–4%), and hypoxemia. Yanik et al. [7] retrospectively assessed 444 patients who underwent bronchoscopy after HSCT and reported that complications developed in 3.6% of patients; 1.8% had low oxygen saturation, 1.7% had bleeding, 0.2% had low blood pressure, and 2% required mechanical ventilation within 2 days. Our results are consistent with the previous studies. In our study, 4 of 5 patients in whom oxygen desaturation did not recover had severe infection (aspergillosis or cytomegalovirus pneumonia), suggesting that their prolonged oxygen desaturation was likely a consequence of severe lung disease rather than a complication of bronchoscopy.

Our study identified use of midazolam to be a favorable factor for reducing the risk of prolonged oxygen desaturation. Midazolam was used as the main agent for sedation during bronchoscopy at our institution starting from the beginning of 2011. Sedation with midazolam improves patients’ comfort and increases their ability to tolerate the procedure without significant hemodynamic changes or respiratory depression [8–10]. Our study shows that the results of the previous studies are applicable to bronchoscopy in patients with malignant hematologic disorders.

Hemoptysis occurred in 35 patients but was not serious except in 1 patient who was intubated just after bronchoscopy and had prolonged oxygen desaturation. TBLB significantly increased the risk of hemoptysis, whereas use of midazolam reduced the risk. The reason why use of midazolam reduced the risk of hemoptysis is not clear, though we speculate that a comfortable procedure as a result of sedation with midazolam might play an important and protective role. On the other hand, platelet count ≤20 × 10^3^/μl and platelet transfusion were not risk factors. The NICE guideline from the British Thoracic Society note that bronchoscopies without biopsy could be safely performed in patients with platelet counts > 20 × 10^3^/μl [11]. In our hospital, patients with platelet count ≤20 × 10^3^/μl received platelet transfusion before bronchoscopy based on this guideline. Weiss et al. investigated bronchoscopies in 58 patients with thrombocytopenia and found complications in 7 (12%) but the complications were serious in only 1 patient [12]. Faiz et al. surveyed 1711 bronchoscopies and found that platelet transfusion was performed in 90.6% of patients with platelet counts of < 10 × 10^3^/μl and 55.5% with platelet counts of 10 × 10^3^ to < 20 × 10^3^/μl [13]. Bleeding occurred in 1.1% of the bronchoscopies but platelet transfusion did not significantly reduce the risk.

This study had several limitations, stemming mainly from its retrospective single-center design. Furthermore, given that midazolam was started in the middle of the study period, we cannot rule out “time-window bias”. In addition, not all patients with hematologic malignancies underwent bronchoscopy. In our hospital, severe disseminated intravascular coagulation and bleeding tendency were contraindication for bronchoscopies, but their definitions were not confirmed. And, the definition of desaturation, we used here, was an unconventional one.

## Conclusions

Bronchoscopy is relatively safe in patients with malignant hematologic disorders provided that respiratory status is monitored carefully after the procedure. Caution and judicious use of sedatives may improve the patient’s procedural tolerance and lower complications. TBLB should be performed with extreme caution in these patients.

## Data Availability

The datasets used and/or analysed during the current study are available from the corresponding author on reasonable request.

## Abbreviations

HSCT: Hematopoietic stem cell transplantation
AML: Acute myeloid leukemia
MDS: Myelodysplastic syndromes
TBLB: Transbronchial lung biopsy
